# Staging TEVAR after FET — an exception or the rule?

**DOI:** 10.1007/s12055-023-01611-7

**Published:** 2023-10-28

**Authors:** Luca Di Marco, Chiara Nocera, Silvia Snaidero, Francesco Campanini, Francesco Buia, Luigi Lovato, Giacomo Murana, Davide Pacini

**Affiliations:** 1 Cardiac Surgery Unit, IRCCS, Azienda Ospedaliero-Universitaria Di Bologna, Via Massarenti 9, 40138 Bologna, Italy; 2 Radiology Unit, IRCCS, Azienda Ospedaliero-Universitaria Di Bologna, Bologna, Italy; 3 Department of Medical and Surgical Science, DIMEC, University of Bologna, Bologna, Italy

**Keywords:** Aortic arch; Frozen elephant trunk technique; TEVAR, Hybrid, Outcomes, Reintervention

## Abstract

**Purpose:**

Frozen elephant trunk (FET) was born as an ideal one-step procedure to treat complex arch and descending thoracic aorta pathology. It was then proved that it frequently needs reintervention, which can often be performed by thoracic endovascular aortic repair (TEVAR) extension since FET provides a safe proximal landing zone. We hereby describe our experience in TEVAR extension after FET, its main indications, technique, and outcomes.

**Methods:**

Between 2007 and 2022, 371 patients underwent FET at our center. Of these, 119 needed TEVAR extension. Some required more than one TEVAR, with a total of 154 procedures. The preoperative characteristics, indications, and outcomes were analyzed retrospectively.

**Results:**

Of 154 TEVAR procedures, 15 were performed in an urgent setting. Mean time from FET to TEVAR was 22,2 ± 28,73 months. Two patients died in the operating room; no others died during the hospital stay. Survival after 1, 2, 5, and 10 years was 96.2%, 93.9%, 90.1%, and 70.5% respectively. There was no statistically significant difference in the rates of TEVAR extension for patients in which a Thoraflex™ vs E-vita™ graft was used, nor for zone 2 vs zone 3 anastomosis and stent length.

**Conclusion:**

Though TEVAR extension is often required after FET, it is a safe and effective procedure with excellent post-operative outcomes in the short-, mid-, and long-term and allows successful treatment of complex aortic pathologies. Rigorous and specialized follow-up after FET is central to identify the right moment to intervene.

## Introduction

When frozen elephant trunk (FET) was introduced in the early 2000s, it represented a revolution in the treatment of complex thoracic aorta pathology by combining open arch surgery and endovascular descending thoracic aorta repair. Though there is no definite consensus on indications for FET, it can be used in chronic aneurysm of the distal arch and thoracic aorta, acute, or chronic type B dissection when endovascular treatment is contraindicated and acute or chronic type A aortic dissection with a primary entry tear in the distal aortic arch or proximal descending aorta when a second step procedure in the downstream aorta can be anticipated [1].

The use of FET has also allowed a process of “proximalization” of the distal anastomosis, which is now often performed in Ishimaru zone 2 rather than zone 3. This allowed easier surgical technique, reduced visceral ischaemia times and no difference in post-operative outcomes [2]. Though shorter, more proximal stent grafts reduce the risk of spinal cord injury [3], some authors linked zone-2 anastomosis to higher risk of secondary aortic intervention [4].

While FET may allow successful single-stage approach in many patients, with good rates of false lumen thrombosis at follow-up [5], it also creates a viable proximal landing zone for secondary thoracic endovascular aortic repair (TEVAR). The need for TEVAR after FET is a common occurrence, with a reported incidence in literature of up to 22% [6], though this percentage varies broadly among centers. This kind of two-stage approach has been proven to be safe and effective [7]. As a second step after complex open aortic surgery, TEVAR showed better post-operative outcomes than open redo of the descending aorta [8]. TEVAR was also linked to lower operative mortality [9] and better quality of life [10] than open descending thoracic aorta surgery.

Though in some patients, TEVAR is performed as an intended completion, in most cases indication is aortic diameter progression, followed by endoleaks and dSINEs.

Our institution is a high-volume aortic center, with a vast experience in FET. At the moment, we have performed 383 FET procedures, of which 371 were considered in these analyses since they were performed before the end of 2022. In this manuscript, we are going to describe our 15-year experience in TEVAR extension after FET. The aim of this study is to describe the short- and long-term outcomes of TEVAR extension after FET and, secondly, to identify factors linked to the need for TEVAR.

## Patients and methods

From 2007 to 2022, we performed FET procedure in 371 patients. Among them, 119 patients underwent TEVAR extension (31.9%). In some cases, more than one extension was executed, with a total of 154 procedures.

Inclusion criteria were all patients who underwent FET at our center in an elective or urgent setting, aged 18 years or over. Exclusion criteria were underage patients and refusing TEVAR extension.

## Surgical technique

Treatment of complex arch and descending thoracic aorta pathology can be considered a multi-step procedure including FET, specialized follow-up, and one or more TEVAR extension.

## Step one: FET procedure

In 2003, Karck et al. [11, 12] first described the frozen elephant trunk technique using a custom-made stent-graft for the treatment of aortic arch and descending aorta aneurysm or chronic dissection [13]. Our experience started in 2007 and since then we performed 383 FET procedures, 371 of which from 2007 to 2022. FET was used to treat acute aortic dissections, chronic dissections, aortic aneurysms and residual dissections. Preoperative planning and correct sizing of the stent-graft portion of the hybrid prosthesis is crucial in order to obtain high rates of false lumen thrombosis at follow-up in aortic dissections [14]. Our surgical technique was previously described [15]. The most frequent arterial cannulation sites at our institution are right axillary artery, brachiocephalic trunk, and right carotid artery, while femoral artery cannulation is only performed in selected cases when other sites are unavailable or for very complex redo surgery. The strategy of cerebral protection is based on selective cerebral perfusion, as described by Kazui et al. [16], with moderate hypothermia during circulatory arrest. The supraortic vessels are reimplated separately [17]. The island technique for supraortic vessel reimplantation is very rarely performed nowadays, while it used to be more common in the early days of FET.

In cases of high risk of spinal cord ischeamia [18], we use cerebrospinal fluid drainage, which is set up and positioned the day before the operation.

In our database, E-vita™ prosthesis was used in 180 patients, Thoraflex™ prosthesis in 190, though the latter is more frequently used in recent years, while most E-vita™ prosthesis were implanted in the first years.

## Step two: follow-up and indication for TEVAR

**Fig. 1:**
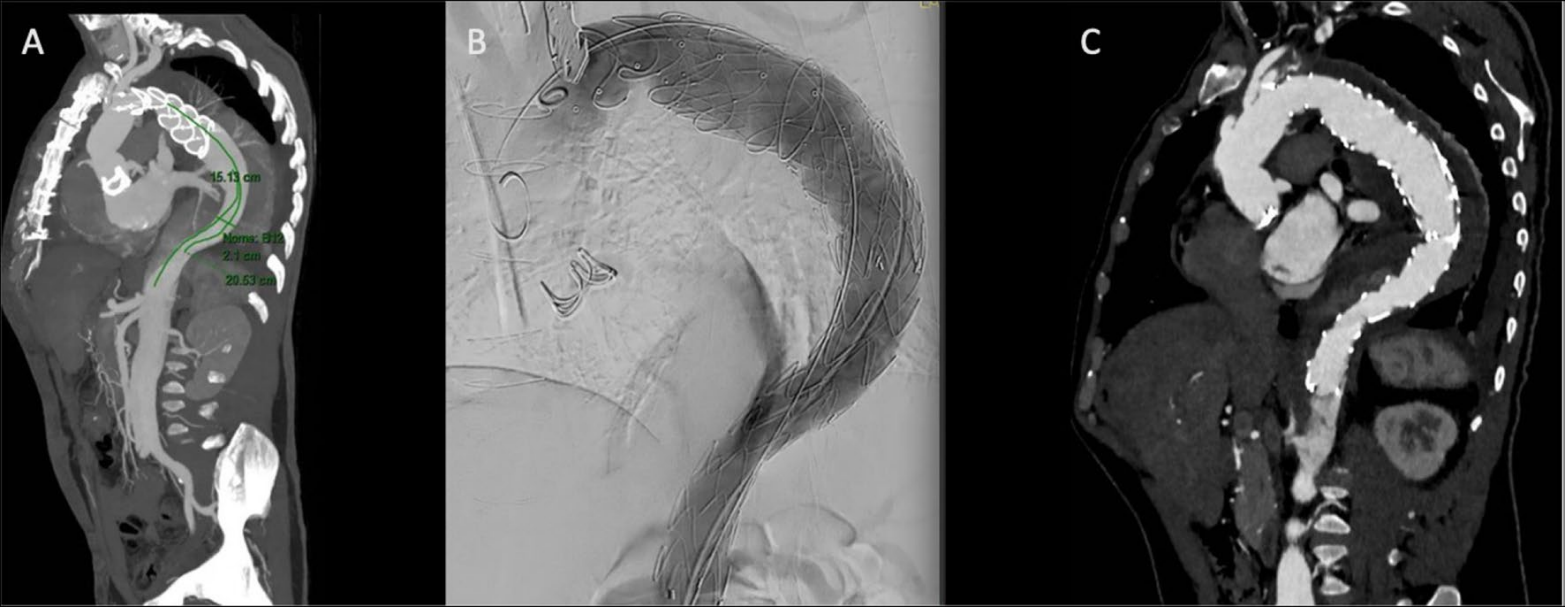
TEVAR extension after FET for residual dissection. **A**: Preoperative CT scan and planning for endovascular extension. **B** Angiographic control after TEVAR. **C** Postoperative CT scan*TEVAR, thoracic endovascular aortic repair; FET, frozen elephant trunk; CT, computed tomography*

All FET patients are evaluated with routine angio-CT scans about 1 week after surgery, then 3 months or sooner if needed. They then undergo routine follow-up with angio-CT or angio-MRI scans and outpatient clinic visits every 6 to 12 months. The follow-up CT scans evaluation and preoperative planning are performed by a multidisciplinary team which includes cardiac surgeons who are experts in aortic pathology and dedicated radiologists who specialize in aortic imaging and endovascular treatment (Fig. 1). The strict follow-up enables us to intercept the patients who need a second-stage procedure with TEVAR extension in a short time. At our institution, when TEVAR extension is indicated, patients undergo preoperative cardiac and renal assessment.

**Fig. 2:**
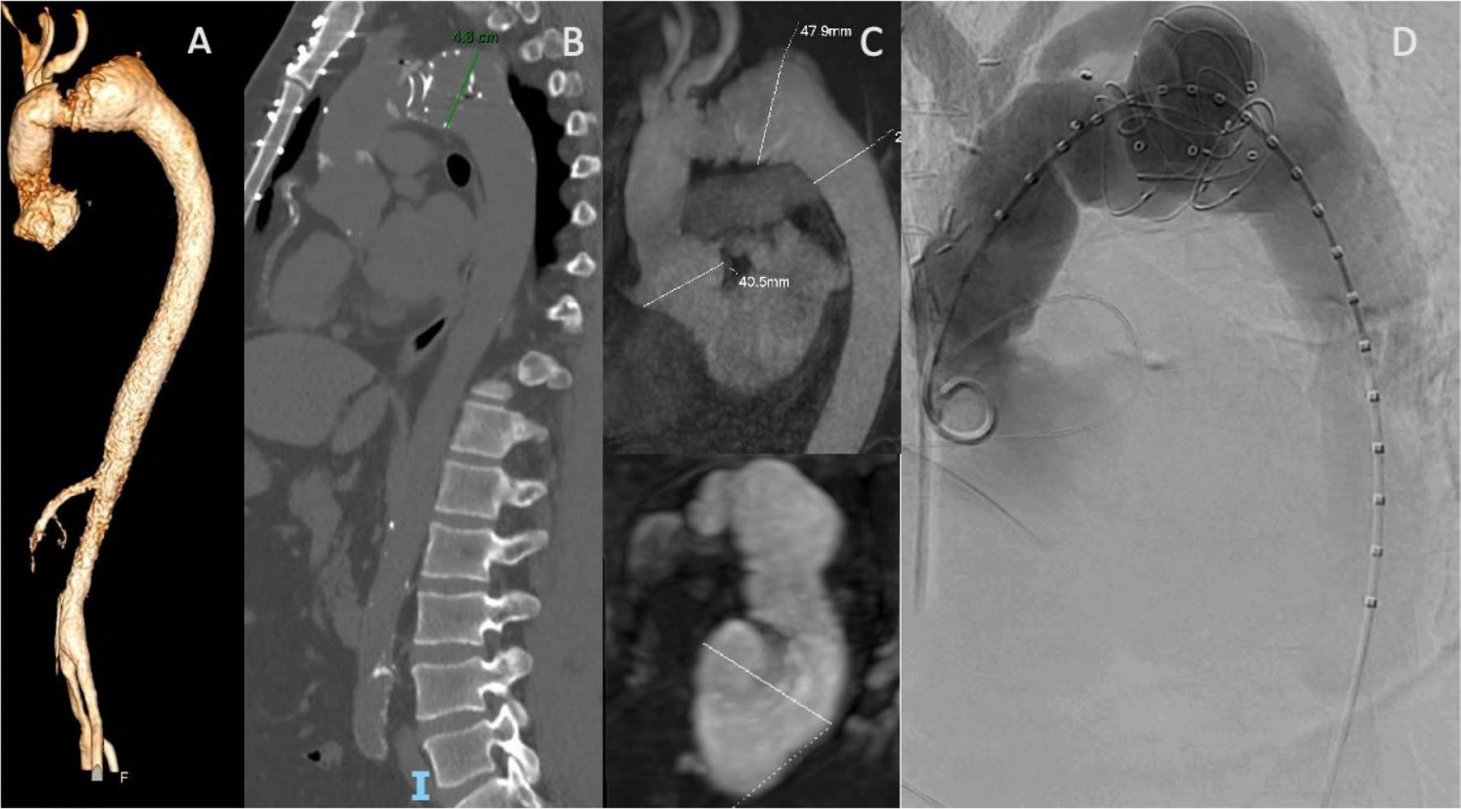
An interesting case of TEVAR extension 6 years after FET. After the patient missed follow-up for 2 years, a control MRI was performed (the patient had recently received a kidney transplantation for Alport syndrome). Malpositioning of the hybrid prosthesis due to proximal migration was found, with the prosthesis not covering the aortic isthmus, which appeared to be dilated (**A**, **C**). A CT-scan without contrast confirmed the finding (**B**). **D** Angiographic aspect before TEVAR. *TEVAR, thoracic endovascular aortic repair; FET, frozen elephant trunk; MRI, magnetic resonance imaging; CT, computed tomography*

**Fig. 3:**
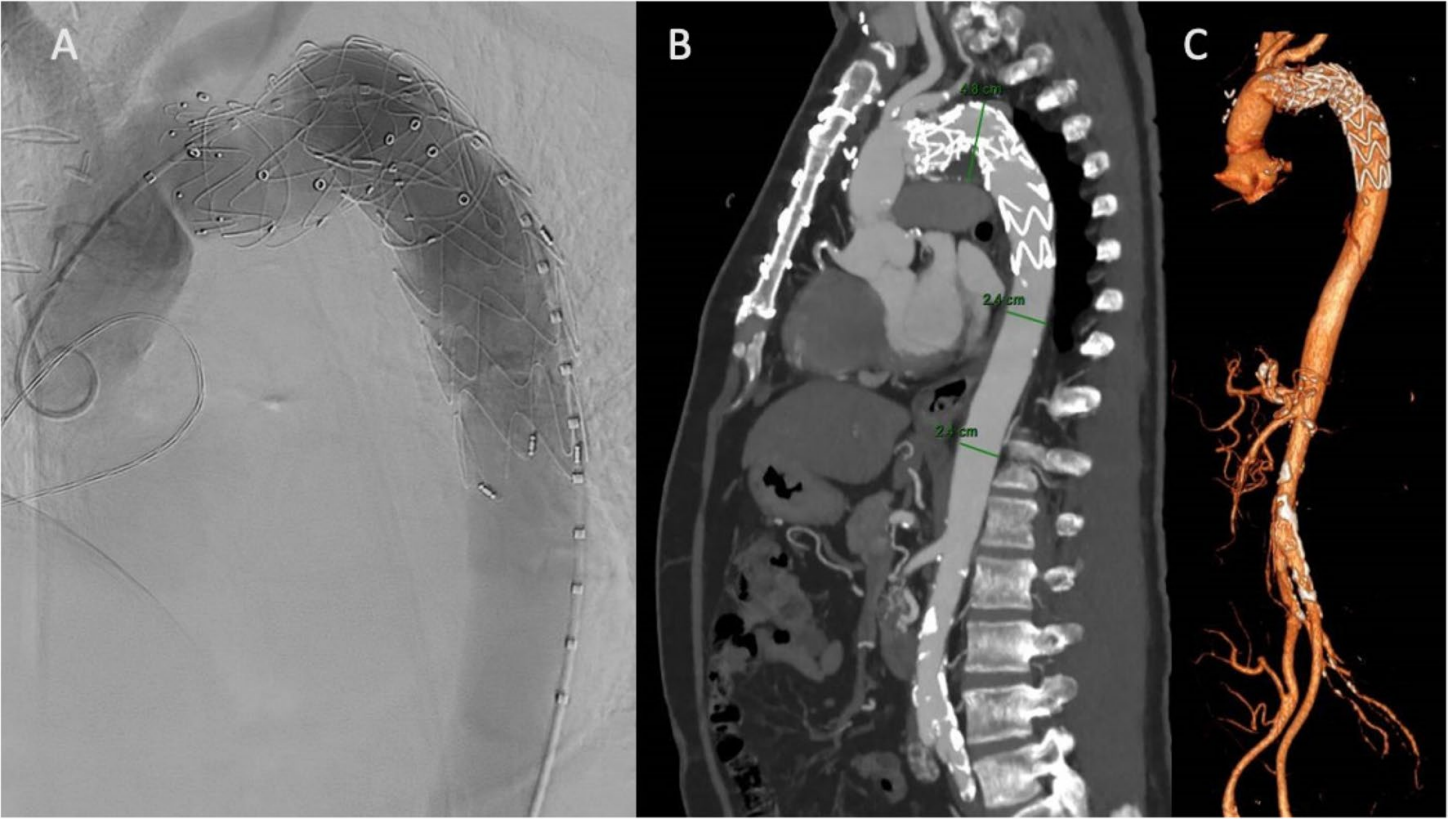
Results of TEVAR in the same patient as Fig. 2. **A** Angiographic control. **B** and **C** Post-operative CT-scan. *TEVAR, thoracic endovascular aortic repair; CT, computed tomography*

As previously stated, most patients who undergo TEVAR extension after FET do so for either progression of the baseline pathology or stent-graft related complications such as dSINEs or various types of stent graft malfunction (Figs. 2, 3). Detailed data on indications can be seen in Table 1.

**Table 1:** Surgical indication and timing for TEVAR extension

Indications:	
• Residual dissection	16/154 (10.4%)
• Distal SINE	43/154 (27.9%)
• Distal end degeneration	22/154 (14.3%)
• Aneurysmal increase	18/154 (11.7%)
• EL	48/154 (31.2%)
▪ EL Ia	2/48 (4.2%)
▪ EL Ib	18/48 (37.5%)
▪ EL II	14/48 (29.1%)
▪ EL III	11/48 (22.9%)
▪ EL IV	3/48 (6.2%)
• Malpositioning in the false lumen	1/154 (0.6%)
• Pseudocoartation/incomplete expansion	6/154 (3.9%)
Urgent TEVAR	15/119 (12.6%)
Mean time for TEVAR	22,2 ± 28,73 months
Mean FU time	56.4 ± 45.75 months

*SINE, stent induced new entry; EL, Endoleakm; TEVAR, thoracic endovascular aortic repair; FU, follow-up*

It is central to have an experienced team of professionals who can identify the right moment to treat each patient based not only on aortic diameters and growth rate but also on the morphology of the lesion. Follow-up time should also be personalized on the patients’ profile, CT-scans findings, and risk factors in order to tailor the treatment on the case.

## Step three: TEVAR

Of the 371 patients who underwent FET, 119 needed at least one TEVAR extension. Mean time from FET to TEVAR was 22.2 ± 28,73 months.

Nowadays, TEVAR are performed by a multidisciplinary team of cardiac surgeons and interventional radiologist in a state-of-the-art hybrid operating room. The most used access is the femoral artery.

Most cases were elective TEVARs, while urgent procedures represented 12.6% of cases. We considered urgent TEVARs to be those performed during the hospital stay or in an emergent setting. Most were extension during the hospital stay due to important false lumen patency at the 1-week control CT-scan, while emergent ones were performed in patients who were admitted to the hospital for chest pain after FET with CT-scan showing increase of aortic diameters or signs of impending rupture.

The intraoperative mortality was 1.7% due to two cases of aortic rupture in the operative room. One of these patients actually went into cardiac arrest for aortic rupture before the procedure started.

## Step four: secondary endovascular reinterventions after TEVAR

In some cases, patients may need more than one TEVAR extension due to complex and diffuse aortic pathology. In our population, 27 patients (22.7%) needed more than one TEVAR, with a total number of secondary TEVAR procedures of 35. In this subgroup, most patients presented aortic dissection as the base pathology.

## Study design and statistical analysis

This is a single-center retrospective study. We collected data on patients who underwent TEVAR extension after FET in our center from 2007 to 2022. All data was collected from clinical reports, pre-operative, post-operative, and follow-up CT scans and outpatient clinic visits. Some required more than one TEVAR, with a total of 154 TEVAR extensions. All variables were expressed either as numbers and percentages or median and standard deviation. IBM SPSS Statistics 27 was used to perform all statistical analysis. Survival at follow-up and freedom from TEVAR were calculated using the Kaplan–Meier method. Univariate analysis was then performed in order to identify factors for TEVAR reintervention at follow-up.

The study was approved by our internal review board (D.P., G.D.G.), which waived the need for inform consent form due to its retrospective nature.

## Results

Patients’ preoperative characteristics are summarized in Table 2. Mean age was 61.5 years, most were men (85.7%). Hypertension was the most frequent cardiovascular risk factor (83.1%), followed by chronic obstructive pulmonary disease (10.1%), diabetes (5%), and chronic kidney disease (3.3%). Marfan syndrome was present in 9 (7.6%) patients. The baseline pathology was represented by aortic dissection in most patients (79.8%, 95 patients of which 27 with acute aortic dissection) and aortic aneurysm in the remaining cases (20.2%).

**Table 2:** Preoperative patients’ characteristics (*n* = 119)

Mean age	61,5 ± 10,8
Male gender	102/119 (85.7%)
Aneursysms	24/119 (20.2%)
Dissections	95/119 (79.8%)
Chronic kidney disease	13/119 (10.9%)
Hypertension	99/119 (83.1%)
Chronic obstructive pulmonary disease	12/119 (10.1%)
Diabetes	6/119 (5%)
Marfan syndrome	9/119 (7.6%)
Thoraflex™ prosthesis	64/119 (53.8%)
E-vita prosthesis	55/119 (46.3%)
Zone 2 distal anastomosis	45/119 (37.8%)
Zone 3 distal anastomosis	74/119 (62.2%)

Mean time from FET to TEVAR extension was 22,2 ± 28,73 months. The most frequent indications for extension were Endoleaks (EL) (31.2%) and dSINEs (27.9%). In particular, the most frequent type of endoleak was EL I b (37.5%), followed by EL II (29.1%), EL III (22.9%), EL IV (6.2%), and Ia (4.2%). In order of frequency, other indications for TEVAR extension were distal end degeneration (14.3%), aneurysmal increase (11.7%), residual dissection (10.4%), pseudocoartation/incomplete expansion (3.9%), and a case of stent graft malpositioning in the false lumen (0.6%).

During the first procedure, Thoraflex™ and E-Vita™ prosthesis were used in 53.8% and 46.3% of the patients who then underwent TEVAR extension, and zone 3 was more frequent than zone 2 anastomosis (62.6% vs 37.8%).

## Primary endpoints

The primary endpoints of the study included in-hospital and follow-up mortality, as well as need for further TEVAR or open surgery at follow-up.

Post-operative outcomes after TEVAR extension of FET are summarized in Table 3. In-hospital mortality was 1.7%, because of two patients who died in the operating room due to aortic rupture. No patients died after TEVAR during the hospital stay.

**Table 3:** Post-operative outcomes

Need for further TEVAR procedure	27/119 (22.7%)
In hospital death (intaoperative)	2/119 (1.7%)
Death at follow up	17/119 (14.3%)
• Aortic death	5/17 (29.4%)
• Other	12/17 (70.6%)
Need for further open surgery	26/119 (21.8%)
Spinal cord ischaemia	0/119
Acute kidney injury	6/119 (5%)
Bowel ischaemia	3/119 (2.5%)
Surgical revision for femoral artery bleeding	4/119 (3.4%)

*TEVAR, thoracic endovascular aortic repair*

**Fig. 4:**
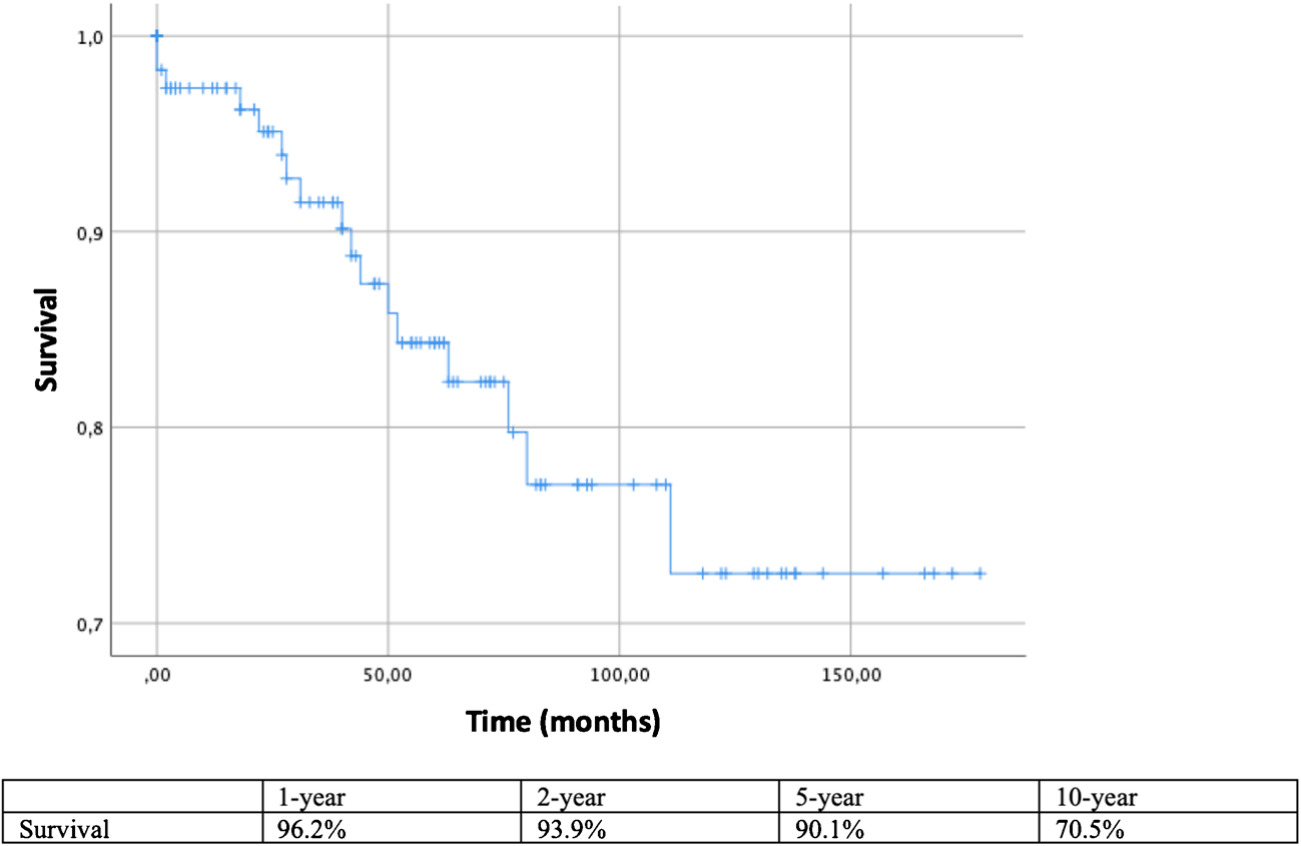
Survival after TEVAR extension of FET. *TEVAR, thoracic endovascular aortic repair; FET, frozen elephant trunk*

Mean follow-up time was 56.4 ± 45.75 months. Death at follow-up was present in 17 (14.3%) patients, 5 of which were due to aortic causes. Survival after 1, 2, 5, and 10 years was 96.2%, 93.9%, 90.1%, and 70.5% respectively (Fig. 4. Patients at risk 88, 79, 67, 17).

After the first TEVAR, 27 patients needed further TEVAR extension, 26 patients needed further open surgical procedures.

**Fig. 5:**
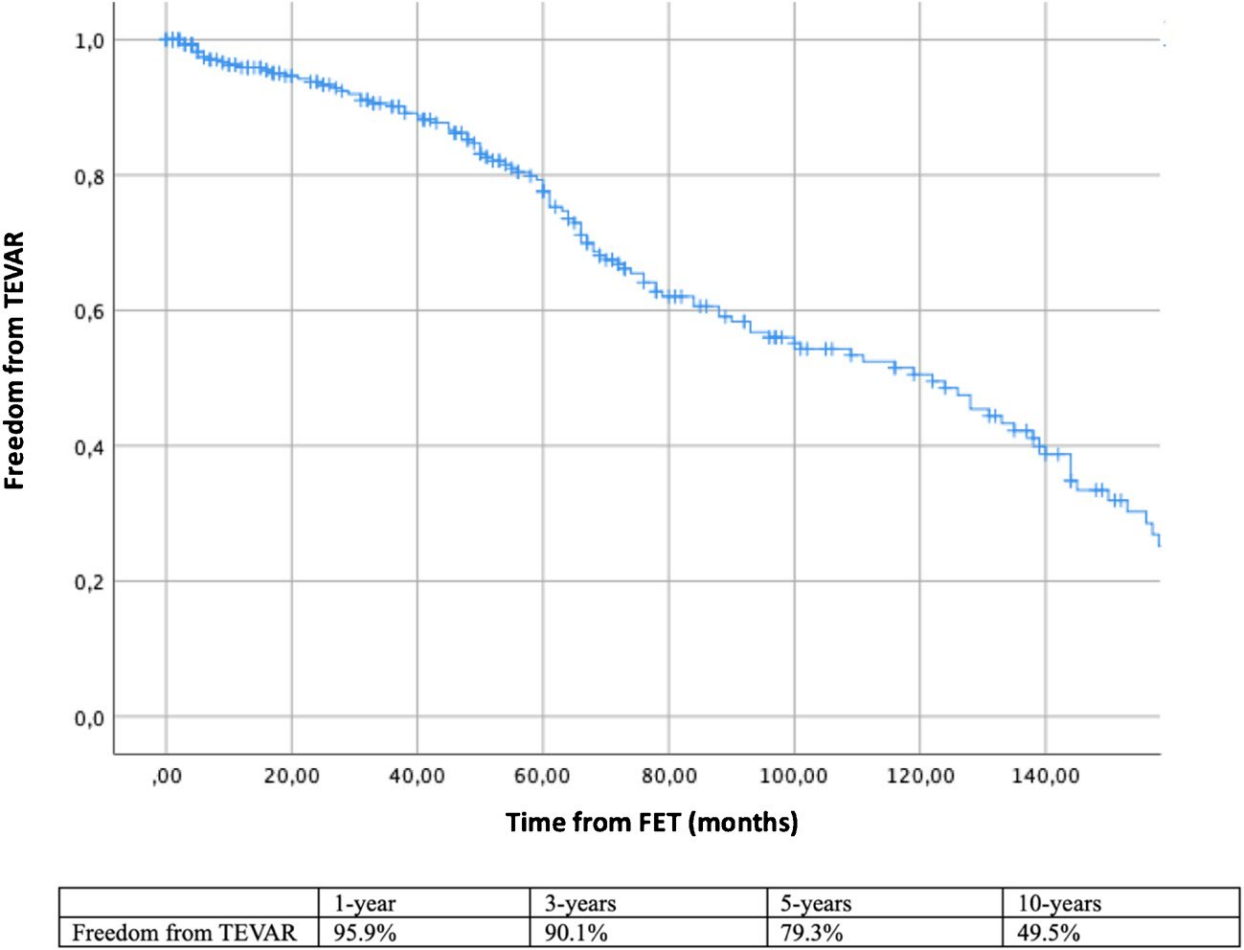
Freedom from TEVAR after FET. *TEVAR, thoracic endovascular aortic repair; FET, frozen elephant trunk*

Freedom from TEVAR after FET at 1, 2, 5, and 10 years of 95.9%, 90.1%, 79.3%, and 49.5% respectively (Fig. 5. Patients at risk 229, 192, 140, and 50).

Event-free survival (combined event of death, TEVAR and surgical redo) at 1, 5, and 10 years was 83.2%, 34.5%, and 20.6% respectively (patients at risk 305, 79, 26).

## Secondary endpoints

Secondary endpoints included complications such as spinal cord injury, bowel ischemia, acute kidney injury, and bleeding requiring reopening.

No patients showed spinal cord injury, while bowel ischemia was present in 3 (2.5%) cases. Acute kidney injury was a complication in 6 [5%] patients, of whom 3 presented preoperative chronic kidney disease. Surgical revision for bleeding of the femoral access was required in 4 patients (3.4%).

## Factors for TEVAR extension

Most of the patients who needed TEVAR extension presented aortic dissection as the baseline pathology, and dissection patients needed TEVAR more frequently than aneurysms (35.4% vs 23.3%), though with no statistically significant difference.

There was also no significant difference in the rates of TEVAR extension for patients in which a Thoraflex™ vs E-vita™ graft was used. The same goes for 2 vs zone 3 anastomosis and stent length (Table 4).

**Table 4:** Statistical univariate analysis of factors linked to TEVAR extension

	Total number	TEVAR extension	
Aneurysms	103	24 (23.3%)	-
Dissections	268	95 (35.4%)	-
Aneurysms vs dissections			*p* = 0.971
E-Vita™ prosthesis	181	55 (30.4%)	-
Thoraflex™ prosthesis	190	64 (33.7%)	-
E-vita™ vs Thoraflex™			*p* = 0.961
Zone 2 anastomosis	152	45 (29.6%)	-
Zone 3 anastomosis	219	74 (33.8%)	-
Zone 2 vs zone 3			*p* = 0.346
100 mm stent grafts	183	63 (34.4%)	-
130 mm stent grafts	38	9 (23.7%)	-
150 mm stent grafts	83	26 (31.3%)	-
160 mm stent grafts	67	21 (31.3%)	-
Stent length			*p* = 0.761

*TEVAR, thoracic endovascular aortic repair*

## Discussion

When FET was first introduced, it was thought to be a single-step alternative to the classic ET, potentially combining the surgical and endovascular part of the treatment of complex aortic arch and descending thoracic aorta pathology [19]. This was particularly exciting for surgeons as the second step procedure after ET was performed less than needed as many patients were lost to follow-up or refused intervention because of the psychological impact of the first surgery, with high mortality rates between the open and endovascular step [20].

As time went on, studies reported high rates of TEVAR extension [6, 21]. The perspective then changed, and it was argued that though FET could not always be a one-step solution, one of its main advantages was providing a safe proximal landing zone for subsequent TEVAR extension, and so it should be considered an effective part of a multiple step procedure [22]. It can even successfully be used with this purpose in complicated type-B aortic dissection with involvement of the aortic arch when TEVAR is not a feasible option [23].

In our experience, 31.9% of patients who underwent FET then needed TEVAR extension. This is consistent with percentages reported by other high-volume aortic centers in literature [6, 11].

We had the chance to study a huge population of patients who underwent FET and to follow these patients through the years extensively and in a rigorous manner, thus analyzing all the following surgical and endovascular steps. What we learned from this is that patients with such complex aortic pathology benefit from being monitored in specialized aortic centers in which surgeons can take care of all their possible needs at follow-up, with both endovascular and open surgical strategies available. Just like 2021 ESC Guidelines for valvular heart disease focused on the need for specialized Heart Valve Centers, in which all kinds of treatment form valve disease are performed [24], the same concept also applies to aortic pathology [25]. It has been proven that specialized centers improve outcomes and reduce mortality in all kinds of surgery [26] in general, and also in aortic surgery in particular [27].

Our data showed low rate of urgent TEVAR and excellent post-operative outcomes. We believe this is due to both careful preoperative planning and to strict follow-up which is tailored to the patients’ condition, risk factors, and CT-scan findings. This allows us to identify the right moment for TEVAR extension. Timing has proven to be crucial in determining outcomes of TEVAR in uncomplicated type B aortic dissection [28]. We believe the same goes for TEVAR after FET, so follow-up is central for getting good results.

Baseline pathology likely plays a role in the likeliness of TEVAR reintervention, with rates of extension in aortic dissections higher than that in aneurysms. In our analysis, however, no statistical difference was found between the two conditions.

It is interesting to compare results of FET with the two prosthesis commercially available in Europe, the Thoraflex™ and e-vita™ hybrid stent grafts [29]. The E-vita™ Open Plus (Jotec GmbH, Hechingen, Germany) was the first commercially available one. In 2012, a new kind of hybrid prosthesis was introduced by Vascutek, the Thoraflex™ hybrid device (Vascutek, Terumo, Inchinnan, Scotland, UK). Good results have been reported with both grafts [30–32]. Some studies showed lower rates of reintervention after FET in patients in whom E-Vita™ graft was used [33]. We have previously reported how, in our experience, the use of the E-vita™ prosthesis was significantly linked to higher incidence of dSINEs [34], probably due to the higher radial force exerted by the graft on the aorta. However, in this analysis, the use of E-vita™ prosthesis has not proven to be linked to higher TEVAR extension at follow-up in general (*p* = 0.923).

The classical ET required a zone 3 anastomosis, which can be technically challenging and is linked to the risk of complications such as recurrent nerve palsy [35]. When FET was first introduced, it was performed with a zone 3 anastomosis. Later, literature started to support the proximalization on the distal anastomosis, which was now performed in zone 2. This allowed reduced visceral ischaemia times, lower rates of recurrent nerve palsy [2], spinal cord injury, and reopening for bleeding [36]. Some authors even suggested further proximalization of the distal anastomosis, which can be performed in zone 1 or even zone 0 [37]. However, some studies reported higher rates of secondary aortic interventions when a zone-2 anastomosis is performed, especially when shorter stent grafts (100 mm) are used [4]. While in the first years of our surgical experience, the distal anastomosis was always performed in zone 3, zone 2 anastomosis is now the standard in most FET procedures at our institution. Our analysis showed no difference in the incidence of TEVAR after FET between zone 2 and zone 3 anastomosis (*p* = 0.565), nor in different stent lengths (*p* = 0.865). Zone 2 anastomosis thus confirms itself to be a safe procedure which can simplify aortic arch surgery and reduce complications.

## Study limitations

The study is limited by its retrospective nature. Furthermore, since it considered patients over a 15-year period, it is influenced by the changes in the surgical technique over this time span (for example the shift from zone 3 to zone 2 anastomosis), so the population is heterogenous.

## Conclusions

Though FET has failed to confirm itself as a successful single-step procedure, its value as a first step in the treatment of complex aortic arch and descending thoracic aorta pathology is undiscussed. Our data confirmed that TEVAR after FET is a safe and effective procedure, especially when it is performed in an elective setting. Rigorous follow-up in specialized aortic centers is mandatory in all patients who undergo aortic arch surgery and collaboration with interventional radiologists can enrich the surgeons’ perspective in the setting of both intended extension for residual dissection or aneurysm, and TEVAR for complications such as ELs and dSINEs.

## Data Availability

The data that support the findings of this study are not openly available due to reasons of sensitivity and are available from the corresponding author upon reasonable request. Data are located in controlled access data storage at Cardiac Surgery Unit of Policlinico di S.Orsola, University of Bologna.

## References

[1] Shrestha M, Bachet J, Bavaria J, Carrel TP, De Paulis R, Di Bartolomeo R (2015). Current status and recommendations for use of the frozen elephant trunk technique: a position paper by the Vascular Domain of EACTS. Eur J Cardiothorac Surg.

[2] Leone A, Di Marco L, Coppola G, Amodio C, Berardi M, Mariani C (2019). Open distal anastomosis in the frozen elephant trunk technique: initial experiences and preliminary results of arch zone 2 versus arch zone 3. Eur J Cardiothorac Surg.

[3] Preventza O, Liao JL, Olive JK, Simpson K, Critsinelis AC, Price MD (2020). Neurologic complications after the frozen elephant trunk procedure: a meta-analysis of more than 3000 patients. J Thorac Cardiovasc Surg.

[4] Liebrich M, Charitos EI, Schlereth S, Meißner H, Trabold T, Geisbüsch P (2021). The zone 2 concept and distal stent graft positioning in TH 2–3 are associated with high rates of secondary aortic interventions in frozen elephant trunk surgery. Eur J Cardiothorac Surg.

[5] Di Eusanio M, Armaro A, Di Marco L, Pacini D, Savini C, Martin Suarez S (2011). Short- and midterm results after hybrid treatment of chronic aortic dissection with the frozen elephant trunk technique. Eur J Cardiothorac Surg.

[6] Haensig M, Schmidt A, Staab H, Steiner S, Scheinert D, Branzan D (2020). Endovascular repair of the thoracic or thoracoabdominal aorta following the frozen elephant trunk procedure. Ann Thorac Surg.

[7] Kreibich M, Berger T, Walter T, Potratz P, Discher P, Kondov S (2022). Downstream thoracic endovascular aortic repair following the frozen elephant trunk procedure. Cardiovasc Diagn Ther.

[8] Kim T, Martin TD, Lee WA, Hess PJ, Klodell CT, Tribble CG (2009). Evolution in the management of the total thoracic aorta. J Thorac Cardiovasc Surg.

[9] Stone DH, Brewster DC, Kwolek CJ, LaMuraglia GM, Conrad MF, Chung TK (2006). Stent-graft versus open-surgical repair of the thoracic aorta: mid-term results. J Vasc Surg.

[10] Dick F, Hinder D, Immer FF, Hirzel C, Do D-D, Carrel TP (2008). Outcome and quality of life after surgical and endovascular treatment of descending aortic lesions. Ann Thorac Surg.

[11] Meisenbacher K, Osswald A, Bischoff MS, Böckler D, Karck M, Ruhparwar A (2022). TEVAR following FET: current outcomes of rendezvous procedures in clinical practice. Thorac Cardiovasc Surg.

[12] Karck M, Chavan A, Hagl C, Friedrich H, Galanski M, Haverich A (2003). The frozen elephant trunk technique: a new treatment for thoracic aortic aneurysms. J Thorac Cardiovasc Surg.

[13] Okita Y (2020). Frozen elephant trunk with frozenix prosthesis. Ann Cardiothorac Surg.

[14] Hoffman A, Damberg ALM, Schälte G, Mahnken AH, Raweh A, Autschbach R (2013). Thoracic stent graft sizing for frozen elephant trunk repair in acute type A dissection. J Thorac Cardiovasc Surg.

[15] Di Marco L, Murana G, Fiorentino M, Amodio C, Mariani C, Leone A (2018). The frozen elephant trunk surgery: a systematic review analysis. Indian J Thorac Cardiovasc Surg.

[16] Kazui T, Inoue N, Yamada O, Komatsu S (1992). Selective cerebral perfusion during operation for aneurysms of the aortic arch: a reassessment. Ann Thorac Surg.

[17] di Marco L, Pantaleo A, Leone A, Murana G, Di Bartolomeo R, Pacini D (2017). The frozen elephant trunk technique: European association for cardio-thoracic surgery position and bologna experience. Korean J Thorac Cardiovasc Surg.

[18] Katayama K, Uchida N, Katayama A, Takahashi S, Takasaki T, Kurosaki T (2015). Multiple factors predict the risk of spinal cord injury after the frozen elephant trunk technique for extended thoracic aortic disease. Eur J Cardiothorac Surg.

[19] Di Bartolomeo R, Pacini D, Savini C, Pilato E, Martin-Suarez S, Di Marco L (2010). Complex thoracic aortic disease: single-stage procedure with the frozen elephant trunk technique. J Thorac Cardiovasc Surg.

[20] Safi HJ, Miller CC, Estrera AL, Villa MA, Goodrick JS, Porat E (2007). Optimization of aortic arch replacement: two-stage approach. Ann Thorac Surg.

[21] Kreibich M, Berger T, Rylski B, Chen Z, Beyersdorf F, Siepe M (2020). Aortic reinterventions after the frozen elephant trunk procedure. J Thorac Cardiovasc Surg.

[22] Kreibich M, Berger T, Rylski B (2021). The frozen elephant trunk: a one-stage, two-stage or even three-stage treatment?. Eur J Cardiothorac Surg.

[23] Weiss G, Tsagakis K, Jakob H, Di Bartolomeo R, Pacini D, Barberio G (2015). The frozen elephant trunk technique for the treatment of complicated type B aortic dissection with involvement of the aortic arch: multicentre early experience. Eur J Cardiothorac Surg.

[24] Vahanian A, Beyersdorf F, Praz F, Milojevic M, Baldus S, Bauersachs J (2022). 2021 ESC/EACTS guidelines for the management of valvular heart disease. Eur Heart J.

[25] Isselbacher EM, Preventza O, Hamilton Black J 3rd, Augoustides JG, Beck AW, Bolen MA, et al. 2022 ACC/AHA guideline for the diagnosis and management of aortic disease: A report of the American Heart Association/American College of Cardiology Joint Committee on clinical practice guidelines. Circulation. 2022;146:e334–482. 10.1161/CIR.0000000000001106 PMC9876736 36322642

[26] Birkmeyer JD, Siewers AE, Finlayson EVA, Stukel TA, Lucas FL, Batista I (2002). Hospital volume and surgical mortality in the United States. N Engl J Med.

[27] Da Cunha SM, Filho JDF, Aguzzoli C, Souza LD, Rösler ÁM, Lucio EA (2014). Centro de Tratamento da Aorta: A especialização reduz complicações e mortalidade. Braz J Cardiovasc Surg.

[28] Xie E, Yang F, Liu Y, Xue L, Fan R, Xie N (2021). Timing and outcome of endovascular repair for uncomplicated type B aortic dissection. Eur J Vasc Endovasc Surg.

[29] Harky A, Fok M, Bashir M (2020). Which is the optimal frozen elephant trunk? A systematic review and meta-analysis of outcomes in 2161 patients undergoing thoracic aortic aneurysm™ surgery using e-vita open plus hybrid stent graft versus thoraflex hybrid prosthesis. Braz J Cardiovasc Surg.

[30] Di Bartolomeo R, Murana G, Di Marco L, Alfonsi J, Gliozzi G, Amodio C (2019). Is the frozen elephant trunk frozen?. Gen Thorac Cardiovasc Surg..

[31] Iafrancesco M, Goebel N, Mascaro J, Franke UFW, Pacini D, Di Bartolomeo R (2017). Aortic diameter remodelling after the frozen elephant trunk technique in aortic dissection: results from an international multicentre registry. Eur J Cardiothorac Surg.

[32] Di Bartolomeo R, Di Marco L, Armaro A, Marsilli D, Leone A, Pilato E (2009). Treatment of complex disease of the thoracic aorta: the frozen elephant trunk technique with the E-vita open prosthesis. Eur J Cardiothorac Surg.

[33] Berger T, Weiss G, Voetsch A, Arnold Z, Kreibich M, Rylski B (2019). Multicentre experience with two frozen elephant trunk prostheses in the treatment of acute aortic dissection. Eur J Cardiothorac Surg.

[34] Murana G, Costantino A, Campanini F, Fiaschini C, Buia F, Mariani C, et al. Distal stent graft-induced new entry (dSINE) after frozen elephant trunk: a scoping review. Cardiovasc Diagn Ther. 2023;13:408–17. 10.21037/cdt-22-234 PMC10423728 37583692

[35] Hirano K, Tokui T, Nakamura B, Inoue R, Inagaki M, Hirano R (2020). Impact of the frozen elephant trunk technique on total aortic arch replacement. Ann Vasc Surg..

[36] Choudhury RY, Basharat K, Zahra SA, Tran T, Rimmer L, Harky A (2021). “Proximalization is Advancement”—zone 3 frozen elephant trunk vs zone 2 frozen elephant trunk: a literature review. Vasc Endovasc Surg.

[37] Tan SZCP, Lopuszko A, Munir W, Adams B, Bashir M (2021). Aortic proximalization—zone 0 versus zone 2: a concept or true challenge?. J Card Surg.

